# Intraoperative Radiation Therapy (IORT) in Gynecologic Cancers: A Scoping Review

**DOI:** 10.3390/cancers17081356

**Published:** 2025-04-18

**Authors:** Evrim Erdemoglu, Stuart A. Ostby, Sanjanaa Senthilkumar, Amanika Kumar, Sujay A. Vora, Longwen Chen, Sarah E. James, Kristina A. Butler

**Affiliations:** 1 Department of Medical and Gynecologic Surgery, Mayo Clinic, Phoenix, AZ 85054, USA; 2 Division of Gynecologic Oncology, Suleyman Demirel University, 32260 Isparta, Turkey; 3 Department of Obstetrics and Gynecology, Mayo Clinic, Rochester, NY 13400, USA; ostby.stuart@mayo.edu (S.A.O.); kumar.amanika@mayo.edu (A.K.); 4 Mayo Clinic Alix School of Medicine, Mayo Clinic, Jacksonville, FL 32224, USA; senthilkumar.sanjanaa@mayo.edu; 5 Department of Radiation Oncology, Mayo Clinic, Phoenix, AZ 85054, USA; 6 Department of Laboratory Medicine and Pathology, Mayo Clinic, Phoenix, AZ 85054, USA; chen.longwen@mayo.edu

**Keywords:** intraoperative radiotherapy, IORT, pelvic exenteration, laterally extended pelvic exenteration, LEER, surgical margin, tumor size, overall survival, disease-free survival, vulvar cancer, vaginal cancer, endometrial cancer, cervical cancer, ovarian cancer

## Abstract

Objective: We aimed to analyze the current literature for IORT in gynecological cancers and summarized clinical outcomes regarding patient selection. Methods: A systematic search was conducted utilizing PUBMED, Embase, and CINAHL to identify studies following PRISMA-ScR guidelines. A PICOS structure was utilized: population: patients with epithelial gynecological cancers; intervention: IORT; C: a comparator was not required, as we aimed to analyze patient selection; outcome: clinical outcomes and overall survival; and S: experimental and quasi-experimental analytical observational studies and descriptive observational studies, excluding case series published in English and limited to the last 10 years. Data extraction was conducted for patient selection, IORT, oncological outcomes, and morbidity. Results: A total of 707 results were identified, and 509 studies were uploaded to Covidence for screening after removing duplications. Of the 21 eligible studies, 9 were included in the final review. The total number of patients included was 348. The studies were retrospective single-institution studies, except for one. There was significant heterogeneity in their design and protocols. IORT was exclusively used for recurrent and advanced stage gynecological cancers adjunct to pelvic exenteration or laterally extended endopelvic resections with variable indications across institutions. The mean number of IORT patients per study was 2.8 per year. Survival rates were variable and dependent on the surgical margin. Endometrial cancer had a favorable outcome compared to vulvar and cervical cancers. Conclusions: Current clinical practice, as demonstrated by the research, is consistent with NCCN guidelines that endorse the application of IORT in instances of recurrent cervical, vaginal, and vulvar malignancies; however, there are no established recommendations for primary tumors. The analysis shows that there are gaps in our knowledge, mainly regarding the status of the margins, the criteria used to choose patients, and the outcomes that are specific to each histology. The standardization of protocols and prospectively powered studies are needed to refine patient selection criteria.

## Simple Summary

This review analyzes how intraoperative radiation therapy (IORT) can be used for gynecologic cancers, focusing on how to choose patients, current clinical practices, and the results of surgery. IORT radiates a targeted tumor bed intraoperatively, which may improve local control and minimize unintended harm to surrounding tissues. A clearly established protocol for patient selection is lacking. Additionally, the efficacy of IORT across different histological subtypes continues to be unclear. The analysis included 21 papers, selecting 9 retrospective studies with 348 patients who underwent IORT in the past decade. IORT has been primarily utilized in cases of recurrent gynecologic malignancies, particularly in instances of central and lateral pelvic recurrences, as well as in the context of pelvic exenteration or laterally extended pelvic resection. Histology-specific results varied, with endometrial cancer exhibiting the highest survival rates, whereas cervical and vulvar tumors demonstrated inferior outcomes. Current clinical practice, as demonstrated by the research, is consistent with NCCN guidelines that endorse the application of IORT in instances of recurrent cervical, vaginal, and vulvar malignancies; however, there are no established recommendations for primary tumors. The analysis shows that there are gaps in our knowledge, mainly regarding the status of the margins, the criteria used to choose patients, and the outcomes that are specific to each histology. There is a need for prospective trials, the standardization of surgical margins, and the incorporation of IORT into minimally invasive surgery.

## 1. Introduction

Intraoperative radiotherapy (IORT) is a specialized technique involving electron beam or high-dose-rate brachytherapy (HDR) to deliver focused radiotherapy to an at-risk tumor bed after surgical extirpation [1]. IORT has the advantage of radiating the field 2–3 times higher than the biological effectiveness of fractioned radiation under direct observation, and application during surgery allows for the protection of other organs by surgical mobilization or shielding [2]. Use is considered when a tumor bed is at risk, and the dose can be adjusted accordingly in patients with previous radiation therapy [1,2]. Since the first description of an IORT procedure performed in 1905 for cervical cancer, patient selection and the definition of an at-risk tumor bed have not been standardized [3]. Although IORT may be utilized for gross positive margins [4], microscopic positive margins, and close margins after surgical extirpation, the benefits of IORT by application and per malignancy are unclear. Herein, we aim to analyze the recent evidence related to patient selection, describe surgical quality, and attempt to standardize indications from publications over the last 10 years (1 January 2014–10 December 2024).

## 2. Methodology (Supplement)

### 2.1. Inclusion Criteria

This scoping review included studies of patients with gynecologic cancers, primarily involving ovarian, endometrial, cervical, vaginal, or vulvar cancers, who have undergone IORT as part of their treatment. A PICO structure with the following aspects was utilized for inclusion in the study: (A) population: studies involving epithelial gynecological cancers who underwent IORT; (B) intervention: studies evaluating the role of IORT in the treatment of gynecological cancers; (C) this review does not require a standard comparator because we seek to analyze the existing literature on patient selection and outcomes; and (D) study design: experimental and quasi-experimental analytical observational studies and descriptive observational studies, excluding case series published in English. Exclusion criteria were studies not focusing on gynecological cancers with overlapping groups or reporting solely on sarcomas and the physical and technical aspects of IORT.

Studies focusing solely on the technical aspects of IORT delivery (e.g., radiation physics and equipment calibration) without reporting clinical outcomes, indications, or complications were excluded. Moreover, studies incorporating IORT for other cancer types (uterine sarcoma), unless gynecologic cancer-specific results were distinctly separated, were excluded.

This review excluded sarcomas due to their tendency to mostly metastasize hematogenously, resulting in distant metastases, rarity, and varied treatment paradigms. Instead, this review focuses on epithelial carcinomas with shared principles of invasion via direct extension, lymphatics, or exfoliation in cases of epithelial ovarian cancers. To achieve a broad search, all study designs were included to allow for a comprehensive review of the existing evidence. Eligible designs encompass experimental and quasi-experimental studies, including randomized controlled trials (RCTs), non-randomized studies of interventions, and interrupted time-series studies. Analytical observational studies, such as prospective and retrospective cohort studies, case-control studies, and analytical cross-sectional studies, were included as well. Descriptive observational designs, excluding case series and individual case reports, were included to offer a thorough overview of the evidence.

### 2.2. Search Strategy/Selection Process and Data Extraction

Three databases were used to identify evidence for screening: PubMed, CINAHL, and Embase. The search was conducted in the period 26 November–10 December 2024 and was limited to studies from the last 10 years and published in English, and the references of relevant systematic reviews were used for backward and forward citation searching using PubMed. Mesh terms using Boolean logic were searched, including intraoperative radiotherapy, ovarian neoplasms, cervical cancer, endometrial cancer, vaginal cancer, and vulvar cancer. Two independent investigators screened all studies identified in our search by the titles and abstracts to determine their eligibility. Disagreements were resolved in a panel meeting including three authors until consensus was reached. The results of the search were managed using Covidence (computer program; Version accessed on 30 July 2024) after transferring them as an XML file using Endnote (The EndNote Team; Version EndNote X9 or EndNote 20; Philadelphia, PA, USA: Clarivate, 2013). Duplicate records were removed. Full text analysis and data extraction were conducted by E.E. and S.K to a data chart template; the list of variables charted are in Table 1, Table 2 and Table 3 and Table S1.

This research aimed to elucidate overall survival (OS), morbidity, and mortality in relation to patient-specific factors to evaluate patient selection. Patient selection included features such as the margin status after resection, tumor diameter, previous therapy types, preoperative indications, tumor origin, and histology. These factors were considered in relation to their impact on overall survival and then evaluated for inclusion by the geographical region, year of publication, study design, and study size. Detailed evaluations of the outcome context included the margin status, tumor diameter in selected patients, previous therapy types, preoperative indications, tumor origin, histology, and toxicities attributable to IORT.

## 3. Results

**Figure 1:**
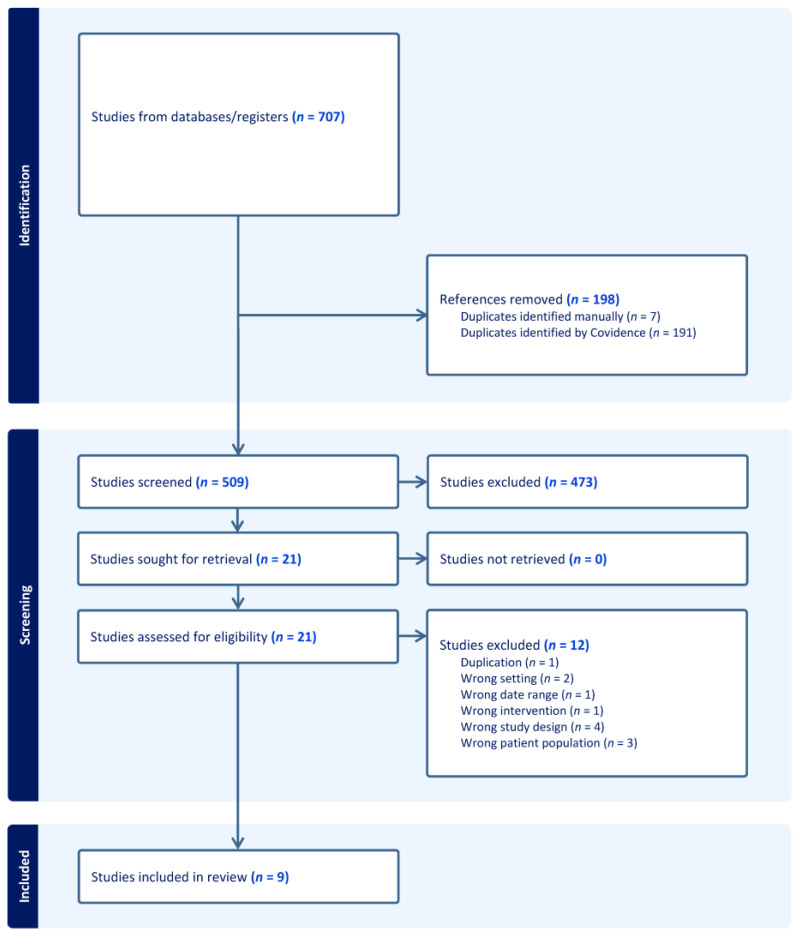
PRISMA-ScR flowchart.

A total of 707 results were identified, and 509 studies were uploaded to Covidence for screening after removing duplications. Of the 21 eligible studies, 9 were included in the final review (8 retrospective studies [5,6,7,8,9,10,11,13] and 1 abstract [12] in the last 10 years) (Figure 1). Commonalities of the eight retrospective papers include their design as retrospective cohort analyses depending on the chart review of patients treated in the previous 10–20 years, yielding a total number of 348 patients receiving IORT. The mean number of IORT patients per study per year was 2.8. There was only one multicenter study (Table 1). Publications were from the United States (*n* = 4), Germany (*n* = 2), Spain (*n* = 2), and Switzerland (single abstract). Study characteristics and extracted data, as well as surgical and oncological outcomes, are shown in Table 1, Table 2 and Table 3 and Supplementary Tables S1 and S2. Study features, patient selection, clinical outcomes, and morbidity are shown in Table 1, Table 2 and Table 3, Tables S1 and S2. Except one study, tumor types were given for each cancer. Endometrial (n = 100), vulvar (*n* = 20), cervical (*n* = 161), vaginal (*n* = 14), and ovarian cancers (*n* = 11) treated with pelvic exenteration and IORT were included. A total of 60–100% of patients had prior radiotherapy. Survival rates were variable and dependent on primary tumor histology and margin status. Time to recurrence had a cutoff value of 12 month or 24 months in different studies. Disease-free and overall survival and recurrence rates are shown in Table 2. The majority of the articles provided pooled outcomes for various tumors.

**Narrative Analysis of Scoping Review**

**Indications and Patient Selection**

IORT was used as a supplementary intervention to surgery for patients with locoregional disease. Surgical treatment most often involved pelvic exenteration or laterally extended pelvic resection.

**Surgical Approach to Advanced Primary and Recurrent Disease and Controversies in Patient Selection**

**Patient Selection for Surgery**

Recurrent gynecologic cancer disseminates through local contiguous, peritoneal, lymphatic, or hematogenous pathways. Locoregional recurrences may manifest as central pelvic recurrences affecting the vulva, vagina, bladder, or rectosigmoid colon. Lateral pelvic recurrence manifests on the pelvic sidewall, affecting the psoas and other pelvic sidewall muscles, ureter, vessels, nerves, and bone. The extent of surgery is usually limited by structures that, if resected, may significantly compromise future function. Treatment alternatives encompass palliative or curative intents, including radiotherapy or surgical intervention. For patients without a history of radiotherapy, the therapeutic alternatives include surgical intervention or radiation therapy. Nonetheless, the NCCN guidelines and others were influenced by low-quality data to recommend treatment for radiation-naïve individuals.

### 3.1. Oligometastatic and Lymph Node Metastasis

Distinct from central and lateral pelvic recurrences at the locoregional tumor origin, solitary and oligometastatic gynecologic cancer recurrences may also be suitable for targeted surgery, radiation, or both [11,14,15,16]. Classifying patients with oligometastatic recurrence into a singular category yielded a highly heterogeneous patient population with significant variability in survival and outcomes. Oligometastatic disease in lymph nodes, both in primary and recurrent contexts, may possess unique biological characteristics and can be managed with surgery. Conte et al. suggested that patients with single or oligometastatic recurrences may be candidates for minimally invasive secondary cytoreductive surgery, particularly when the recurrences are localized in the lymph nodes [17]. Sole et al. explored the outcomes and risk variables for patients with para-aortic lymph nodes related to gynecological malignancies who underwent surgical resection followed by IORT. They reported that the 5-year locoregional control was 79%, disease-free survival (DFS) was 44%, and overall survival was 49% [11].

### 3.2. Primary and Recurrent Settings

No studies in the past decade investigated the role of IORT in primary treatment. Our analysis yielded that prior radiation varies between 60 and 100% (Table S2). NCCN (NCCN Version 2.2025) recommendations indicate that patients with recurrent cervical cancer with a history of prior radiation may have surgical resection and IORT (Category 3), despite the unfavorable prognosis. These principles are extrapolated to centrally recurrent vaginal and vulvar carcinomas. In contrast with guideline suggestions for IORT as a re-irradiation tool, IORT is not limited to settings following prior radiation therapy in our analysis (Table S2); however, the evidence quality is low. Surgical intervention for advanced gynecological malignancies is tailored to the individual and is typically not the primary treatment modality, nor is it commonly used in conjunction with IORT. Only one phase II trial, published in 2011, suggests that radiochemotherapy followed by surgery and IORT might be a feasible option for the primary treatment of locally advanced cervical cancer [18]. More robust data and higher quality studies are needed to clarify the role of IORT in the primary setting.

In 2008, cervical cancer with metastasis to para-aortic lymph nodes was classified as stage IV. It was revised to stage IIIC2 in the 2018 FIGO Cancer Report [19]. Various sites of metastatic lymphatic disease have varied outcomes [19]. Stage IVB cervical cancer may arise from inguinal or other distant lymphatic metastases or hematogenous dissemination. A recent study on stage IVB showed that the use of local surgery or radiotherapy enhances the likelihood of survival [20,21]. Cervical cancer patients with distant lymphatic metastases are thought to have superior overall survival compared to those with hematogenous and disseminated disease. This may be ascribed to the chemoradiosensitivity in low-burden tumors in distant lymph nodes [22,23]. In this patient cohort, surgery may enhance the quality of life by alleviating hemorrhaging, discomfort, and fistulas while increasing the likelihood of locoregional control. NCCN 2025.V2 recommends local resection combined with tailored external beam radiation therapy (EBRT) or local ablative therapy alongside EBRT for managing stage IVB cervical cancer. As mentioned above, IORT is utilized for primary tumor resection, and we speculate that in particular cases of stage IVB cervical cancer, IORT combined with surgical intervention may be considered, and its effects might be evaluated in future research. Similar to cervical cancer, surgical intervention is recommended in some metastatic patients with stage IVB vaginal cancer, wherein IORT may be considered in specific circumstances.

When pelvic exenteration is conducted for primary indications, it can be postulated that IORT may be utilized when margins are microscopically positive or relatively close. However, pelvic exenteration for the treatment of primary tumors remains controversial. Marnitz et al. reported that exenteration is an alternative to primary chemoradiation in patients with histopathologically proven stage IVA cervical carcinoma [24]. The 5-year overall survival in the primary exenteration was statistically higher than the 5-year overall survival of patients who had exenteration for the recurrent setting (52.9% vs. 26.7%). Survival showed a significant correlation with the time interval between primary treatment and recurrence, as well as with the intention of treatment—whether curative or palliative—and with the presence of tumor-free resection margins. The study revealed that factors such as age, the type of exenteration, histologic type, and metastatic spread to pelvic lymph nodes did not have a significant impact on long-term survival. Ungar et al. proposed a 50% survival rate for selected stage IVA cervical cancer patients who underwent primary exenteration. They have also claimed that low rectal anastomosis combined with orthotopic bladder replacement presents a relatively low risk of fistula formation in non-irradiated patients, thereby providing a compelling argument for the improvement of quality of life in a carefully chosen cohort of stage IVA cervical cancer patients undergoing primary exenteration [25]. Additional studies confirm these findings, suggesting that pelvic exenteration represents a valuable option in both recurrent and primary surgical treatments [26,27]. The EORTC-55994 study failed to demonstrate the superiority of neoadjuvant chemotherapy before surgery [28]. Another randomized controlled trial assessing the efficacy and toxicity of neoadjuvant chemotherapy prior to radical surgery in patients with locally advanced squamous cervical cancer did not demonstrate the superiority of neoadjuvant chemotherapy [29]. More studies assessing the impact of neoadjuvant chemotherapy prior to radiotherapy have indicated that it simply increases the toxicity without yielding any improvement in the overall survival rates [30]. Neoadjuvant chemotherapy before surgery may be used to improve the feasibility of a successful operation, does not result in overall survival differences compared to chemoradiation, and has attendant higher short-term morbidity balanced by possibly lower long-term morbidity to 2 years. [28,29,30]. Additionally, recommendations concerning neoadjuvant therapy vary among country-specific guidelines, resulting in a lack of a definitive indication for its use [31].

In IORT-planned patients, Delara et al. demonstrated that preoperative EBRT or chemotherapy was not associated with complete tumor resection or with survival [7]. Sole et al. studied IORT in patients with lymph node metastases, and they reported that in these patient groups, preoperative EBRT improves the locoregional control rate without having any impact on overall survival [11]. Howlett et al. reported that neoadjuvant chemotherapy or immunotherapy increases the perioperative morbidity in patients receiving IORT [9]. The use of chemotherapy and/or immunotherapy after the diagnosis of recurrence, prior to exenteration/IORT, was significantly associated with an increased risk of death within three years following surgery [9]. Perioperative EBRT or neoadjuvant chemotherapy appears ineffective in achieving complete resection and influencing overall survival in patients receiving surgery with IORT [7,8]. It can be argued that this is due to the selection of more advanced disease with larger tumors and patient factors.

The findings can be framed alongside other studies that advocate for adjuvant treatment before radiochemotherapy; notably, the recent INTERLACE study [31] demonstrated that the administration of induction chemotherapy prior to definitive radiochemotherapy yields enhancements in survival. The overall survival rates at three years were 85% for the group receiving induction chemotherapy combined with chemoradiotherapy compared to 80% for the group undergoing chemoradiotherapy alone. Nonetheless, this study encompasses patients with stage IB1-IVA, and the subgroup analyses demonstrating the necessity of examining treatment effects across different stages, as well as the relationship between mortality and survival in relation to the stage, which remain unpublished. An important finding of this study was that a portion of deaths (17–21%) was due to disease progression. Further studies exploring the role of surgery in this subgroup are required.

In the previous Retro-EMRACE non-randomized study investigating the role of image-guided brachytherapy in locally advanced cervical cancer, the results demonstrated that the 3-year and 5-year overall survivals declined to 56% and 42% in stage IIIB compared to 88% and 83% in stage 1B, respectively. This was even lower (32%) in stage 4A patients (*n* = 23) [32]. In EMBRACE I, a prospective observational study examining the role of MRI in image-guided adaptive brachytherapy, the five-year overall survival rates were reported as 64% for stage IIIB (*n* = 190), 52% for stage IVA (*n* = 34), and 61% for stage IVB (*n* = 98) [33]. The results obtained were somewhat comparable to those observed in studies involving external beam radiotherapy followed by brachytherapy, which reported a 5-year overall survival rate of 52.8% to 57.8% [30]. However, the advanced radiotherapy techniques reduced the grade 3–4 toxicities to 14.6%, with most cases occurring in stage III-IV patients [33]. Comparing the curative pelvic exenteration results from retrospective surgical studies to other observational studies of radiotherapy or randomized trials of chemoradiotherapy is not feasible, as these latter studies are not adequately powered to identify any differences in overall survival between primary surgical treatment and alternative methods. Finally, it is important to note that the definitions of palliative and curative exenteration vary across different institutions [24,34].

While there is a controversy in the neoadjuvant treatment of locally advanced cervical cancer, ongoing trials are exploring the potential of immunotherapy, such as KEYNOTE-826, BEATcc, and ENGOT-cx11/GOG-3047/KEYNOTEA18. While discussions of these are out of our scope and these trials are not in the setting of neoadjuvant treatment, these studies yielded that the addition of pemrolizumab, atezolizumab, and bevacizumab in combination with radiation and chemotherapy provides superior results compared to chemotherapy or radiation. Current evidence from these trials suggests the use of immunotherapy in T3 or T4 tumors with any node and no distant metastasis. Additionally, these studies lack a surgical comparison arm [35,36,37].

Primary exenteration has been suggested for various gynecological cancers beyond cervical cancer. According to the findings of Valstad et al., the experience with pelvic exenteration in cases of both primary advanced vulvar cancer and recurrent vulvar cancer indicated that cancer-specific survival rates exceed 60% [38]. It was reported that among patients receiving treatment for primary advanced vulvar cancer, 44% experienced a relapse compared to a 57% relapse rate after exenteration for recurrent vulvar cancer. Most of the recurrences were locoregional [38]. Other studies reported that primary pelvic exenteration for vulvar cancer had worse outcomes: According to the experience at the Sloan Kettering Cancer Center, factors such as an age greater than 62 years (hazard ratio (HR): 2.71; 95% CI: 1.27–5.79), American Society of Anesthesia (ASA) classifications of 3–4 (HR: 3.41; 95% CI: 1.03–11.29), and the presence of vulvar cancer (HR: 4.19; 95% CI: 1.17–14.96) were associated with poorer overall survival in patients with progressive or recurrent gynecologic cancer who underwent pelvic exenteration [39]. However, on multivariable analysis, there were no significant factors associated with worse overall survival. A report from the MD Anderson Cancer Center analyzing pelvic exenterations conducted between 1993 and 2010 indicated that survival outcomes have not markedly improved, even with advancements in technique and patient selection (5-year overall survival = 40%) [40]. Multiple non-modifiable factors at the time of exenteration were associated with poor survival. The study identified that factors adversely affecting overall survival included vulvar primary (*p* = 0.04), positive margins, lymphovascular space invasion (LVSI), positive lymph nodes, and perineural invasion. In the multivariate analysis, both positive nodes and LVSI demonstrated a significant influence on overall survival [40]. It is essential to take into account both modifiable and non-modifiable risk factors prior to planning the operation. Further studies are needed to identify the role of primary pelvic exenteration in various gynecologic cancers.

The curative potential of pelvic exenteration is frequently assessed in relation to mortality and morbidity. Although mortality rates have decreased in recent years from 23% to 2%, severe morbidity continues to affect 21% to 34% of patients undergoing pelvic exenteration [41] with cervical cancer, identified as the most significant risk factor for morbidity. Hospital volume is reported to be another independent factor influencing perioperative mortality. The perioperative mortality rates were observed to be 3.7% in centers with minimal surgical volume, defined as one exenteration per year. In contrast, centers conducting more than one but two or fewer exenterations annually exhibited a mortality rate of 1.4%. Notably, the top decile centers, which performed more than two exenterations per year, reported a mortality rate of 0% (*p* < 0.001) [42]. The risks associated with pelvic exenteration, such as the inability to attain negative surgical margins and the possible complications arising from chemoradiation in advanced disease, which may result in a fistula rate between 22% and 47%, require careful consideration with respect to one another. Furthermore, it is essential to assess individualized risks for local control failure, including factors such as tumor diameter, origin, histology, and lymph node metastasis, within the framework of the ongoing discourse regarding primary exenteration [43,44]. Recognizing the risks associated with failure in exenteration typically pertains to chemoradiation failure and is considered a negative prognostic indicator for patients undergoing chemoradiation [45,46], Therefore, treatment should be tailored to the specific needs of each patient in advanced stages. Treatment options need to be considered in a multidisciplinary tumor board and communicated to the patient and their family throughout the decision-making and treatment process.

In the near future, the assessments regarding morbidity associated with treatment modalities will also include a novel category, especially minimally invasive pelvic exenteration with or without IORT. Our institutional experience with robotic pelvic exenteration, in which we also implement IORT, is likewise progressing. A recent meta-analysis encompassing 11 studies with a total of 264 patients indicated a trend toward improved survival rates associated with minimally invasive exenteration [47], alongside a reduction in morbidity [47,48,49,50,51].

To integrate IORT into surgical procedures for primary tumors, it is essential to document the additional toxicity associated with IORT. This would assist in evaluating the application of IORT in primary disease. Nonetheless, it is not feasible to extrapolate the morbidity associated with IORT and surgical procedures. Furthermore, a majority of the patients documented in the existing literature have undergone prior irradiation. In certain patients with locally advanced disease, the extension of the IORT indication to primary treatment warrants further investigation

### 3.3. Primary Tumor Type and Histology

Prognostic differences exist between various gynecological cancers, irrespective of IORT [13]. The primary tumor and histology are a reflection of molecular characterization and tumor biology, where these are central to determining outcomes [52]. Aside from cervical, vaginal, and endometrial cancers, IORT is not referenced in the 2024 NCCN guidelines. Patients presenting with recurrent isolated vulvar cancer confined to local lymph nodes are managed through external beam radiation therapy (EBRT) or systemic therapy, particularly in cases where prior irradiation has been administered. In cases of vulvar recurrence, surgery followed by radiation is recommended exclusively for patients who are node negative. Some consider ovarian cancer very radiosensitive and would see this as a great target of EBRT. In cases of persistent or recurrent ovarian cancer accompanied by oligometastatic disease, localized radiation is administered as a potential option, especially for palliative care.

Recent publications over the past decade have documented the application of IORT in cases of recurrent vulvar, vaginal, endometrial, or ovarian cancers. Sprave et al. did not observe an OS difference when analyzed by grade or histology but had limited ability to detect differences with only 40 patients [13]. Additionally, the number of vulvar (*n* = 10) and vaginal cancer (*n* = 0) patients was very low (Table 1 and Table 2). Arains et al. reported that recurrent endometrial cancer exhibits the greatest benefit from the combination of IORT, achieving a five-year survival rate of 50%. In contrast, patients with cervical and vulvar cancers demonstrated poorer outcomes, with five-year overall survival rates of 6.4% and 16.7%, respectively, as well as diminished locoregional control [5]. Similarly, Kumar et al. reported that although there was no statistical difference, the overall 3-year survival in cervical cancer patients treated by pelvic exenteration with IORT was worse (41.0% (95% CI: 28.4–59.2%)) than in endometrial cancer patients treated by pelvic exenteration with IORT (58.6% (95% CI: 43.6–78.8%)) [9]. A comparable favorable prognostic trend for endometrial cancer and an unfavorable prognostic trend for vulvar cancer is also noted in pelvic exenteration without IORT; in an analysis of 230 patients who underwent pelvic exenteration for gynecological cancer from 1998 to 2011, Chiantera et al. reported that the overall mortality rate, based on the tumor site at the conclusion of the study, was 75% for vulvar cancer, 57.6% for cervical cancer, 55.6% for vaginal cancer, and 53.6% for endometrial cancer [53].

Cancers of unknown origin in the pelvis may represent another rare indication for IORT [7]. The NCCN guidelines reveal an important deficiency in knowledge pertaining to cancers of unknown origin, particularly restricted to the pelvis. The anatomical sites of the tumor are categorized into the following regions: head and neck, supraclavicular, axillary, mediastinum, lung, inguinal nodes, liver, bone, and brain. Radiotherapy is recommended as an adjuvant treatment following the dissection of a single lymph node exhibiting extra nodal extension or an insufficient dissection. Radiotherapy is exclusively considered for retroperitoneal masses of non-germ cell histology or for supraclavicular nodal involvement in site-specific squamous carcinoma.

### 3.4. Central Recurrence and Pelvic Sidewall Recurrence/Pelvic Exenteration and Laterally Extended Endopelvic Exenteration

The existing guidelines lack clarity regarding patient selection and provide minimal information concerning sidewall involvement, laterally extended endopelvic exenteration (LEER), and IORT. Only one manuscript was identified that compared the effect of IORT in LEER (*n* = 8) [6]. This study involved 32 patients, of whom 11 underwent pelvic exenteration alone, while 13 received exenteration in conjunction with IORT. The median progression-free survival and overall survival for patients with exenteration alone were 33 months and 41 months, respectively. In contrast, those with exenteration plus IORT exhibited progression-free survival and overall survival of 10 months and 10 months, while patients receiving LEER plus IORT had progression-free survival and overall survival of 9 months and 17 months. This research observed that positive surgical margins significantly influence the outcome of IORT. It can be argued that the LEER procedure may demonstrate superiority over standard pelvic exenteration; however, patients undergoing LEER experienced a higher incidence of distant recurrence [6].

IORT is not shown to improve the survival in pelvic sidewall involvement requiring the LEER procedure [6]. A more radical surgical approach should be balanced between morbidity due to a more complex surgical procedure and survival outcomes. More studies are needed exploring the impact of IORT in conjunction with LEER. In our previous publication reporting the results of 80 patients with IORT, sidewall involvement was associated with grade 3+ postoperative complications (OR = 8.80; 95%CI = 1.09–70.86; *p* = 0.04). Other factors increasing morbidity were poor ECOG scores of 2–3 (OR = 18.00; 95% CI = 1.81–178.78; *p* = 0.01) and chemotherapy or immunotherapy prior to surgery (OR = 6.98; 95% CI = 2.03–24.02) [9]. While these were not associated with oncological outcomes, they increased the perioperative morbidity.

### 3.5. Margin Status

Microscopic tumor deposits located beyond the tumor’s edge have long been recognized by pelvic surgeons. In patients with poorly differentiated tumors, the distal spread is typically more extensive and discontinuous when compared to those with well- and moderately differentiated tumors [54]. This mandates intraoperative frozen section evaluation prior to IORT. Margin assessment is conducted intraoperatively, initially through gross evaluation by the surgeon utilizing both digital and visual examinations. The resected specimen, along with additional biopsies from the surgical margin or surgical bed after bulk tumor removal, undergo pathological evaluation with frozen section analysis.

Arians et al. reported that the margin status had no effect on overall survival [5]. The R0 rate was 41.7% among a total of 36 patients. The overall survival rates at 1 and 5 years were 65.3% and 21.7%, respectively. However, the criteria for determining the margin status and the application of frozen section analysis were not clearly articulated in this study. In an alternative study that determined the margin status to be insignificant [6], the criteria for the decision-making process regarding IORT remain unclear; it is noted that IORT was left to the discretion of the attending surgeon and was dependent upon the margin status or clinical suspicion of positive margins. The definition of clinical suspicion was not given, and data regarding the number of patients undergoing IORT based on clinical suspicion were not provided. Furthermore, it was reported that the pathologic evaluation lacked reliability in the cohorts. This study further concluded that margin status does not significantly influence progression-free survival and determined that the effects of IORT on margin status could not be ascertained.

In contrast to these two studies, the publication that defined surgical margins and employed frozen sections to assess the margin state indicates that the margin status is the most critical aspect when evaluating IORT. A multicenter study involving 80 patients from the Mayo Clinic in Minnesota, Arizona, or Florida, conducted from June 2004 to May 2021, revealed that patients with positive resection margins had inferior survival outcomes, with an overall three-year survival rate of 9.2% (95% CI = 1.5–57.8%), in contrast to 57.6% (95% CI = 45.8–72.5%) for margin-negative patients. In this cohort, surgical resection achieved negative margins in 72.5% of patients, although microscopically positive margins were observed in 12 patients (15.0%), and the presence of residual disease was significantly correlated with mortality within 3 years [9]. Sole et al., in their analysis of 55 patients, classified surgical margins as negative, microscopic, or extensive residual and determined that the margin status is the sole significant predictor influencing overall survival in multivariate analyses [11]. Delara et al. [7] classified surgical margins in 44 patients receiving IORT as negative, microscopic, or macroscopic residual tumors, with frozen section evaluation performed for all patients. Here, 56.8% demonstrated negative surgical margins, with four patients (10.8%) showing no viable tumor, while eleven patients (29.7%) exhibited microscopic disease. In this cohort, the 3-year progression-free survival rates for patients with negative, microscopic, and macroscopic margins were 51.8%, 20.5%, and 0%, respectively (*p* = 0.006), while the 3-year overall survival was 62.9%, 20.0%, and 0%, respectively (*p* = 0.035). These findings correspond with previously documented results [4,55,56].

A recent study involving 32 patients who underwent IORT and surgery, with assessment of grossly negative margins via frozen section to ensure microscopically negative margins, revealed a 5-year progression-free survival of 40.9% in patients with microscopic residual tumors, compared to 9.1% in those with macroscopic residual tumors. Additionally, the 5-year OS rates were 77.3% for microscopic and 54.5% for macroscopic residual tumors [8]. The findings from these studies reflect the results of pelvic exenteration and may suggest that if significant macroscopic residual tumors remain post-extirpation, IORT does not enhance outcomes. The importance of the surgical margin is also implied in other tumors. These findings align with head and neck malignancies, where IORT yields superior overall survival and progression-free survival in R0 resections, followed by R1 resections, in contrast to those with substantial macroscopic residuals. Research involving 1291 patients who had pelvic exenteration for locally advanced rectal cancer found that multivariable analysis showed the resection margin and nodal status as significant predictors of overall survival [54]. Margin positivity, after risk adjustment for patient and tumor factors, is one such metric that has been proposed as a quality indicator to measure hospital performance in rectal cancer [54].

The preoperative plan and patient selection should prioritize those who will derive the greatest advantage from IORT through full resection to negative or, at minimum, narrow margins. There are multiple potential reasons for this, not least of which is the non-standardized application of IORT in the treatment of positive margins amongst heterogeneous patient populations. At present, we need a definition of a negative margin by tumor site and histology.

To this end, in the Sloan Kettering definition, positive margins are defined as microscopically positive and close margins within 1 mm of the inked margin of resection [10]. In a previous study [57] to determine the effect of distance to the closest negative margin in pelvic exenteration, close and distant negative margins were defined as <3 mm and ≥3 mm from malignancy to the nearest surgical margin, respectively. Distant margins were associated with improved OS (*p* = 0.0054) and progression-free survival (*p* = 0.0099) compared to close margins. Median survival was 32 months (95% CI: 14–62) for close margins and 111 months (95% CI: 42–166) for distant margins. After adjusting for other prognostic factors, patients with distant margins had a significantly decreased risk of all-cause mortality (HR = 0.39; 95% CI = 0.19–0.78; *p* = 0.008) and progression (HR = 0.48; 95% CI = 0.23–0.99; *p* = 0.04) compared to positive margins. No significant differences in overall survival or progression-free survival were observed between close and positive margins. These findings are also in accordance with other pelvic cancers such as rectal cancer, where it is reported that narrow and exposed surgical margins had an almost equal impact on local recurrence and poor overall survival after pelvic exenteration [58,59,60].

It is generally accepted that all patients must have a confirmed recurrence and undergo evaluation for R0/R1 resection in a multidisciplinary tumor board collaboration which may aid in better patient selection associated with negative margins, overall survival, and lower morbidity. Patients with gross disease prior to IORT should not be regarded as optimal candidates. During surgery, a rigorous strategy must be employed not only to ensure negative surgical margins but also to prevent tumor fragmentation. Our study of the literature from the past decade suggests that abandoning IORT in cases of gross macroscopic disease may be considered if there is particularly a concern for increased toxicity from IORT. Patients with macroscopic surgical margins can be assessed in the context of palliative surgery without increasing morbidity by IORT. On the other hand, the overall survival of margin-negative patients receiving IORT is encouraging (Table 2 and Table 3), and the indications for IORT can be extended beyond only microscopic positive cases to include selected margin-negative individuals.

In centrally situated tumors with microscopic positive or close margins, re-excision or IORT is a viable choice. Nonetheless, IORT may be more essential in cases of negative narrow or microscopic positive margins when the resection plane is positioned laterally. In exenterations that engage lateral planes, a thin margin may be unavoidable due to constraints posed by bone, nerves, or arteries. The involvement of the lateral pelvic sidewall and lymph nodes elevates the chance of positive surgical margins. The alternative, more morbid procedure in this context is composite resection; however, to the author’s knowledge, no study has compared the superiority of composite resections to LEER with IORT. However, as previously mentioned, data on IORT and LEER are limited, and these individuals may not be ideal candidates. Ensuring negative margins in the intended LEER may be of paramount importance. The comprehensive assessment of IORT failure should be conducted in relation to surgical margins, with success and benefits primarily considered in patients with negative margins, followed by those with microscopic positive margins. Although additional benefits are a reward of IORT, the bulk of failures are primarily due to surgical margins.

The surgical margin can be evaluated either by shaving or radial sections taken from where the tumor is grossly closest to the margin [59]. Shave margins are those in which small pieces of tissue are sampled from the periphery of a resection. This is done when the margins are not thought to harbor tumor and certainly, at least, are not grossly noted to harbor tumor. They are a ‘positive or negative’ exercise in that the presence of any tumor, regardless of its amount or location in the tissue, indicates a positive margin. Radial margins, on the other hand, are those taken where one is purposely sampling the tumor and the leading edge of a resection perpendicularly in the same histologic section so that the margin can be seen, and the distance from the tumor to the margin observed. These findings indicate that evaluation for microscopic disease on the margin status is important and should be standardized in studies related to surgery and IORT. After the removal of the gross tumor, a surgeon takes samples of tissue from the resection margins and sends them to pathology for evaluation. Despite the accuracy of frozen sections on surgical margins being high and potentially varying among institutions, there is still the possibility of wrong sampling. New techniques and novel developments such as fluorescence-guided surgery need to be assessed for their impact on the margin status and detection of microscopic disease in previously irradiated patients.

### 3.6. Tumor Size

For the studies included in this review, the tumor size is shown in Table 1. The significance of the tumor size in patients with a planned IORT is highlighted in two of the selected studies; Backes et al. found, in a cohort with a mean tumor diameter of 5–6.5 cm, that an increase in tumor size adversely affected progression-free survival (HR = 1.3; 95% CI = 1.12–1.52; *p* < 0.001) and overall survival (HR = 1.15; 95% CI = 1.05–1.26; *p* = 0.003) [6].

Delara et al. showed that individuals with a median tumor diameter of 4.4 cm were more likely to achieve complete surgical resection, whereas those with a tumor diameter of 6.5 cm had a higher incidence of microscopic or gross remaining surgical margins [7]. A tumor diameter exceeding 3–5 cm is identified as a negative prognostic factor in pelvic exenteration without IORT, even with the absence of a definitive threshold for tumor size [49,56,61]. As much as the tumor diameter affects resectability, there seems to be evidence that with an increasing size, there are lesser survival outcomes.

### 3.7. Distant Metastasis and Advanced Locoregional Disease

There is consensus on the published material that distant metastasis is an absolute contraindication to IORT with surgery. An increasing tumor size negatively impacted progression-free survival (HR = 1.3; 95% CI = 1.12–1.52; *p* = 0.001) and overall survival (HR = 1.15; 95% CI = 1.05–1.26; *p* = 0.003) [6]. Preoperative PET-CT is valuable for finding distant metastases, while MRI can assess locoregional disease. Diagnostic laparoscopy may be utilized before the operation to detect disseminated peritoneal and lymphatic disease [62]. Koehler et al. reported that diagnostic laparoscopy prior to pelvic exenteration revealed unresectable illness in 48% of patients scheduled for pelvic exenteration. The procedure was abandoned due to intraperitoneal disease (55%), para-aortic lymph node metastases (30%), and extensive unresectable pelvic sidewall involvement (15%) [63].

### 3.8. Time to Recurrence

The treatment-free interval or time to recurrence is a significant prognostic indicator for pelvic exenteration, regardless of the use of IORT. The IORT trials in this analysis indicated that a longer disease-free interval correlated with a reduced mortality risk within three years [9]. An interval of less than 6 months is classified as persistent cancer. A time delay of less than 24 months after primary tumor diagnosis is considered a negative prognostic indicator for pelvic exenteration with intraoperative radiation therapy (IORT) [11]. On the contrary, Sprave et al. reported that the disease-free interval between the initial diagnosis and first recurrence (<12 vs. ≥12 months) and the disease-free interval to IORT (<12 vs. ≥12 months) did not significantly affect overall survival [13]. The findings of Sprave et al. can be re-evaluated in the context of previously published studies [24,64,65] on pelvic exenteration, which imply that the time to recurrence cutoff should be 24 months. Survival exhibited a significant correlation with the duration between primary treatment and recurrence: 16.8% five-year survival within 1–2 years, 28% between 2 and 5 years, and 83.2% after more than 5 years (*p* = 0.0105) [24].

### 3.9. IORT Technique and Discussion of Which Patients Need Additional Perioperative Chemo/Radiotherapy

If external beam radiation therapy for curative intent achieves a dose of 6000–7000 cGy or higher, this may pose safety concerns for the patient, and IORT can be considered. External beam therapy is palliative because doses greater than 50 Gy in 25–28 fractions often cannot be delivered safely, and this dose reaches 60 Gy if the margins are microscopically or grossly positive. IORT in these groups is an alternative to external beam therapy but in order to achieve the desired outcome, the margins should be negative at the end of the surgery [66]. The technique of IORT and size of the applied cone might also have an impact on the oncological outcomes. It should be discussed by the tumor board and after surgical extirpation to confirm it is individualized to the patient. However, it is beyond the aim of our review (Figure 1). The cone size varied between studies as well as the IORT technique. The target area was defined in correspondence with the surgeon and usually included the high-risk area for positive margins with a safety margin of 1 cm [5].

### 3.10. Medically Fit Patients

Preoperative assessment of the patient is crucial to pelvic exenteration and IORT. The preoperative evaluation must encompass a nutritional assessment. Systemic inflammation, hypermetabolism, and involuntary weight loss and muscle loss contribute to malnutrition and increased morbidity in malignancy [67]. Tortorella et al. reported that hemoglobin ≤ 10 g/dL, low albumin levels, diabetes, and two or more comorbidities at presentation were predictors of severe early complications in univariable analysis [68,69]. However, in multivariable analysis, only low hemoglobin and comorbidities at presentation were independent predictors of complications. Parenteral iron therapy can be implemented in the preoperative treatment. Preoperative exercise and lifestyle modifications are shown to improve cardiopulmonary tests and correlated with improved postoperative recovery and decreased hospital stay [70].

In general, age, smoking, and BMI are considered to be important [71,72]. There was one study evaluating the effects of patient factors on the perioperative morbidity in patients treated with IORT; it was found that patients with poor ECOG (score 2–3) were more likely to experience grade 3+ toxicity, while age, smoking, and BMI were not associated with postoperative complications. Similarly, a retrospective review of 71 pelvic exenterations at MSKKC revealed that, in univariable analysis, overall survival was adversely affected by an age over 62 years, a diagnosis of vulvar cancer, and an ASA classification of 3 or 4. In the multivariable study, no factors adversely affected overall survival [39]. Maggioni et al. published a study of 106 patients receiving pelvic exenteration for gynecologic cancer, revealing no statistically significant difference in survival across all age groups [56]. There is also controversy regarding BMI. While age, ASA score, preoperative albumin, FIGO stage, surgical complexity, and BMI are used to refine the prediction model for complications after primary debulking surgery for advanced epithelial ovarian cancer [73], the current studies suggest that chronological age and BMI are not a limiting factor for pelvic exenteration. The range of patients included in this analysis was between 25 and 80 years old.

The Charlson Comorbidity Index (CCI) is a widely used clinical tool that predicts a patient’s 10-year mortality risk based on their comorbid conditions. Matsuo et al. reported that a high Charlson score (≥3) was linked to more postoperative adverse events, longer hospital admissions, and increased mortality in a retrospective investigation of 2647 pelvic exenterations for gynecologic malignancies [74].

Nevertheless, a limited sample size and underpowered conclusions may neglect the significance of an albumin level below 3, elevated ASA score, BMI, age, and other variables; hence, a customized strategy for each patient is essential, and a comprehensive preoperative assessment is vital.

## 4. Morbidity and Mortality

The modern overall mortality rate in selected patients for pelvic exenteration is 3.8% [68]. In our analysis, the addition of IORT to exenteration toward overall mortality and morbidity was limited to one study (Table S1). Arians et al. reported the highest death rate: death in 3 patients out of 36 (8.3%) due to hemorrhagic shock [5]. This study suggested that the elevated mortality rate may be attributable to LEER procedures and vascular reconstructions. It is also of note that this study incorporated the lowest number of patients per year (1.2 patients per year) with the highest duration (26 years incorporating different time periods) while other studies incorporated a mean of 2.9 patients per year (range 1.8–4.2). It can be postulated that the limited patient volume over an extended period may influence overall mortality and morbidity. In the other studies included in this analysis, the death rate was 0 in five studies, 2.7% (1/37, anastomosis leakage and sepsis) in one study [7], and 1.3% (1/80) in another study [9]. In the study comparing IORT to perioperative high-dose brachytherapy [10], there were four fatalities among 25 patients (16%) who underwent perioperative high-dose brachytherapy (one due to small intestinal obstruction and three due to pelvic hemorrhage), whereas death linked to IORT was only reported in 1 patient (1/33, 3%). Two additional patients in the IORT group were lost due to ischemic stroke and respiratory sepsis (2/33, 6%).

Grade 3+ toxicity in patients treated with IORT reached up to 23–50%. Grade 3+ toxicity in patients undergoing IORT ranged from 23% to 50%. While the toxicities of intraoperative radiotherapy (IORT) during pelvic exenteration remain indistinguishable from surgery, issues potentially associated with IORT include nerve injury (4.7–11.1%), femoral head necrosis (8.3%), wound complications (22.2%), fistula formation (5%), and other complications (13.9–29%), as well as ureteral stricture.

## 5. Limitations and Strengths of the Present Review

Several limitations of this review are related to the retrospective design of the included studies, and heterogeneity in the study designs and treatment protocols should be acknowledged. The studies were retrospective, and a lack of randomized controlled trials reduces the level of evidence. There was no power analysis of IORT treatment as a reflection of small cohort sizes. IORT techniques and doses were not constant, and the margin definitions were vague.

## 6. Future Directions

Patient selection is paramount for surgery followed by IORT to be successful and requires greater standardization by procedure type. The vulnerable patient population and the presence of advanced disease impose significant challenges on the surgeon, with each decision bearing its own implications. The length of time to generate observational, single-institution cohorts limits our conclusions and likely does not reflect the outcomes available following the implementation of new technologies and additional drug treatments. Multicenter collaborations will be necessary to prospectively determine which patients are most likely to benefit. The art of determining which patients may benefit from surgery and IORT with curative intent is largely still descriptive and in need of standardization and prospective analyses.

## 7. Conclusions

In conclusion, IORT has been primarily utilized in cases of recurrent gynecologic malignancies, particularly in instances of central and lateral pelvic recurrences, as well as in the context of pelvic exenteration or laterally extended pelvic resection. Histology-specific results varied, with endometrial cancer exhibiting the highest survival rates, whereas cervical and vulvar tumors demonstrated inferior outcomes. Current clinical practice, as demonstrated by the research, is consistent with NCCN guidelines that endorse the application of IORT in instances of recurrent cervical, vaginal, and vulvar malignancies; however, there are no established recommendations for primary tumors. The analysis shows that there are gaps in our knowledge, mainly regarding the status of the margins, the criteria used to choose patients, and the outcomes that are specific to each histology. There is a need for prospective trials, the standardization of surgical margins, and the incorporation of IORT into minimally invasive surgery.

## Figures and Tables

**Table 1:** Summary of studies on IORT in gynecological malignancies. The data highlight the availability of IORT and heterogeneity in patient cohorts, treatment settings, and the variation in IORT applications.

Study	Geographical Location	Start Date	Finish Date	Total *n*	Duration of Study	IORT Volume per Year	Vulvar Cancer (*n*)	Cervical Cancer (*n*)	Endometrial Cancer (*n*)	Vaginal Cancer (*n*)	Other Primary (*n*)	Median Tumor Size, cm (Range)
Arians, 2016 [5]	Germany	2002	2014	36	12	3	6	18	12	0	0	N.G.
Backes, 2014 [6]	USA	2000	2012	32	12	2.67	3	21	0	8	0	5–6.5 (0–13)
Delara, 2021 [7]	USA	2004	2019	37	15	2.47	1	8	20	2	2 + 4 (ovarian + sarcoma)	5 (1–12)
Foley, 2016 [8]	USA	1994	2011	32	17	1.88	0	21	6	4	1 (ovarian)	1.5 (0.6–5.2)
Howlett, 2024 [9]	USA	2004	2021	73	17	4.29	0	45	35			N.G.
Jablonska, 2021 [10]	Spain	1985 and 2000	1996 and 2015; IORT and PHDRB, respectively	IORT: *n* = 33; PHDRB: *n* = 25; total is *n* = 58	* 1985–1996 and 2000–2015; total is 26 yrs	2.23						5 (0.5–12.5)
Sole, 2015 [11]	Spain	1997	2012	35	15	2.33	0	18	14	0	3 (ovarian)	6 (2–10)
Pagano, 2023 * [12]	Switzerland	2014	2022	30	8	3.75		19	3		8 (7 sarcoma + 1 ovarian)	N.G.
Sprave, 2024 [13]	Germany	2010	2022	40	12	3.33	10	11	10	0	9 (6 sarcoma + 3 ovarian)	N.G.

* = congress poster and the only study utilizing IORT in a primary setting in 20% of the cases; PHDRB: perioperative high-dose radiation brachytherapy; IORT: intraoperative radiation therapy; N.G.: not given.

**Table 2:** Features of patient groups and study cohorts in IORT.

Study	Total *N*	Age (Range)	Comparation/Cohort	IORT Dose	R0 Resection	Histologic Type	Organ Involvement	Lymph Node Metastasis	Prior Radiation	Prior Surgery	Median Time to IORT	Mortality
Arians, 2016 [5]	36	50.4 (27–76)	No cohort; overall survival compared based on cancer type	15 Gy (10–18 Gy)		Squamous: 19 (52.8%); adenocarcinoma: 13 (36.1%); serous: 3 (8.3%); mucinous: 1 (2.8%)	20 (55.6%)	13	24/36	35/36	55.9 (months)	
Backes, 2014 [6]	32	PE + IORT = 59; LEER + IORT = 59; only surgery = 48	Cohorts based on surgery type; cohorts are PE + IORT, LEER + IORT, and only surgery (control)	PE + IORT: 15 Gy (15–20); LEER + IORT: 17.5 Gy (10–17.5)	PE + IORT: 5 (38%); LEER + IORT: 1 (13%); only surgery (control): 4 (36%)	All cohorts with IORT: squamous: 24 (75%); adenocarcinoma: 1 (3%); serous: 1 (3%); other: 3 (9%)	N/A	N/A	PE + IORT: 13 (100%); LEER + IORT: 8 (100%); only surgery: 11 (100%)	N/A	PE + IORT: <24 m 11 pt (85%); LEER + IORT: <24 m 4 pt (50%); only surgery: <24 m 3 pt (27%)	N/A
Delara, 2021 [7]	Any type of surgery + IORT: *n* = 37; PE + IORT: *n* = 10 PE + 8 CRS (para-aortic LND) + 1 vaginectomy; LEER + IORT: *n* = 18 (13%); only Surgery (control): *n* = 7 (4 only LEER and 3 only PE w.o. IORT)	Any type of surgery + IORT = 62	Cohorts based on surgery type; cohorts are PE + IORT, LEER + IORT, and only surgery (control)	Any type of surgery + IORT: 10–18 Gy	Any type of surgery + IORT: 21 (56.8%)				Any type of surgery + IORT: 37/44 (84%)	Any type of surgery + IORT: 36/44 (82%)	Any type of surgery + IORT: 36 m	Any type of surgery + IORT: 22 (59.5%)
Foley, 2016 [8]	32	54 (32–80)	No cohort; overall survival not compared by groups; instead, survival was observed	13.5 Gy (10–22.5)	0%	12 (37.5%) squamous, 12 (37.5%) adenocarcinoma, 2 (6.3%) sarcoma, 2 (6.3%) carcinosarcoma, and 4 (12.5%) other			28 (87.5%)	76.30%	21 (65.6%)	
Howlett, 2024 [9]	Any type of surgery + IORT = 73; only surgery: 7	56.8 SD 13.7	Cohorts were any type of surgery + IORT and only surgery (control); overall survival compared based on cancer type		72.50%				Any type of surgery + IORT: 76.3%	Any type of surgery + IORT: 76.3%	23.2 m	1 (1.3%)
Jablonska, 2021 [10]	IORT: *n* = 33; PHDRB: *n* = 25; total is *n* = 58	Any type of surgery + IORT = 47 (25–71); perioperative radiotherapy = 57 (28–73)	Cohorts based on radiation type; any type of surgery + IORT and perioperative radiotherapy		Any type of surgery + IORT = 18 (55%); perioperative radiotherapy = 9 (36%)	Any type of surgery + IORT = 24 (73%) squamous, 8 (24%) adenocarcinomas, 1 (3%) clear cell/serous; perioperative radiotherapy = 12 (48%) squamous, 10 (40%) adenocarcinomas, 2 (8%) clear cell/serous, and 1 (4%) other		Any type of surgery + IORT = 6 (18%); perioperative radiotherapy = 10 (20%)	Any type of surgery + IORT = 33 (100%); perioperative radiotherapy = 25 (100%)	Any type of surgery + IORT = 7 (21%); perioperative radiotherapy = 16 (64%)		Any type of surgery + IORT = 22 (66.7%); perioperative radiotherapy = 14 (56%)
Sole, 2015 [11]	35	59 (37–73)	No cohort; all participants received IORT, and survival was observed based on EBRT	12.5 Gy (7.5–15)	18 (51%)	14 (40%) squamous, 21 (60%) adenocarcinomas			14 (40%)			18 (51%)
Pagano, 2023 [12]	30										38.2 m	
Sprave, 2024 [13]	40	58 (26–78)	No cohort; overall survival, local regional control, and distal metastasis were observed	Any type of surgery + IORT: 13.8 Gy (10–18)	24 (60%) confirmed; 11 (27.5%) status unknown/cannot be evaluated	20 (50%) squamous, 9 (22.5%) adenocarcinomas, and 11 (27.5%) other			Any type of surgery + IORT: 27 (67.5%)	Any type of surgery + IORT: 35 (87.5%)	Any type of surgery + IORT: 15 m (5–112)	

PE: pelvic exenteration; LEER: laterally extended endopelvic resection; PHDRB: perioperative high-dose radiation brachytherapy; IORT: intraoperative radiation therapy.

**Table 3:** Oncological outcomes in IORT.

Study	Total *N*	1-Year OS	2- or 3-Year OS	5-Year OS	Median OS	1-Year DFS	2- or 3-Year DFS	5-Year DFS	Locoregional Recurrence	Distant Recurrence
Arians, 2016 [5]	36	Vulvar: 83.3%; endometrial: 83.3%; cervical: 44.5%		Vulvar: 6.4%; endometrial: 50%; cervical: 6.4%	14				18 (50%)	16 (44%)
Backes, 2014 [6]	PE + IORT: 13; LEER + IORT: 8; only surgery: 11	PE + IORT: 50%; LEER + IORT: 63%; only surgery: 83%	PE + IORT: 10%; LEER + IORT: 20%; only surgery: 60%	PE + IORT: 0%; LEER + IORT: 20%; only surgery: 33%	PE + IORT: 17; LEER + IORT: 10; only surgery: 41	PE + IORT: 55%; LEER + IORT: 45%; only surgery: 82%	PE + IORT: 18%; LEER + IORT: 18%; only surgery: 47%	PE + IORT: 0%; LEER + IORT: 18%; only surgery: 33%	PE + IORT: 31%; LEER + IORT: 37.5%; only surgery: 36%	PE + IORT: 38%; LEER + IORT: 62.5%; only surgery: 9%
Delara, 2021 [7]	Any type of surgery + IORT:	Any type of surgery + IORT:	Any type of surgery + IORT:	Any type of surgery + IORT:	Any type of surgery: 52 margin negative		Any type of surgery + IORT: 51.8% (margin neg.), 0% (margin pos.); only surgery (control): 54.8% (margin neg.)		Any type of surgery + IORT: 25 (67.5%); only surgery (control): 0%	Any type of surgery + IORT: 25 (67.5%); only surgery (control): 0%
					+					
	37 IORT	90% (margin negative)	62.9% (margin negative)	50% (margin negative)	10 to 14 margin positive					
				
					only surgery (control):					
	PE +IORT:	85% (margin positive)	0–20% (margin positive)	0% (margin positive)						
	10 PE + 8 CRS (para-aortic LND) + 1 vaginectomy				61 margin negative					
				
	LEER + IORT: 18		Only surgery (control):							
			70.8% (margin negative)							
	Only surgery (control): 7 (4 only LEER and 3 only PE w/o IORT)									
Foley, 2016 [8]				69.70%				30.30%	13 (40.6%)	4 (12.5%)
Howlett, 2024 [9]	73	Endometrial: 87%; cervical: 67%	Any type of surgery + IORT: 48.6%; endometrial: 58.6% (95CI 43.6–78.8); cervical: 41% (95CI 28.4–59.2)		34 m					
Jablonska, 2021 [10]			Any type of surgery + IORT = 19.1% (2 yr); perioperative radiotherapy = 19.1% (2 yr)	Any type of surgery + IORT = 17.8%; perioperative radiotherapy = 17.8%			Any type of surgery + IORT = 17.2% (2 yr); perioperative radiotherapy = 17.2% (2 yr)	Any type of surgery + IORT = 15.5%; perioperative radiotherapy = 15.5%	Any type of surgery + IORT = 18 (54.6%); perioperative radiotherapy = 11 (44%)	Any type of surgery + IORT = 17 (51.5%); perioperative radiotherapy = 7 (28%)
Sole, 2015 [11]	35	85%	55%	49%		85%	50%	44%	21%	
Pagano, 2023 [12]			Any type of surgery + IORT: 58.1% 2 yr				Any type of surgery + IORT: 18.2% 2 yr			
Sprave, 2024 [13]		80%	69%	55%					18 (45%)	9 (22.5%)

OS: overall survival; DFS: disease free survival; PE: pelvic exenteration; LEER: laterally extended endopelvic resection; IORT: intraoperative radiation therapy.

## Supplementary Materials

The following supporting information can be downloaded at https://www.mdpi.com/article/10.3390/cancers17081356/s1: Table S1. Toxicity in IORT; Table S2. IORT was utilized in patients without prior radiation and with prior radiation.
