# Supplementary material for: Intraoperative Radiation Therapy (IORT) in Gynecologic Cancers: A Scoping Review
Source: Cancers (Basel). 2025 Apr 18;17(8):1356. doi: 10.3390/cancers17081356 (PMC12025500; doi:10.3390/cancers17081356)
Supplement: Supplementary file 1 [file cancers-17-01356-s001.zip › cancers-3504953-supplementary.pdf]

Supplementary File

Table S1: Toxicity in IORT

Study	Total N	Death	Nerve Damage	Femoral Neck Necrosis	Fistula	Wound Complication	Other
**Arians, 2016**	36	3	4	3	0	8	5
**Backes, 2014**	21		1 (4.7%)				6 (29%)
**Delara, 2021**	n/a	n/a	n/a	n/a	n/a	n/a	n/a
**Foley, 2016**							46.9% grade 3/4 toxicity
**Howlett, 2024**	73	1 (1.3%)					16 (20.3%)
**Jablonska, 2021**		Any type surgery + IORT = 22 (66.7%) Perioperative Radiotherapy = 14 (56%)					
**Sole, 2015**		Any type surgery + IORT – 18 (51%)					
**Pagano, 2023**							
**Sprave, 2024**					Any type surgery + IORT – 5 (12.5%)	Any type surgery + IORT – 6 (15%)	Any type surgery + IORT – 8 (20%)

IORT = Intraoperative radiation therapy

Table S2: IORT was utilized in patients without prior radiation and with prior radiation.

Study	Prior Radiation (n)
**Arians, 2016**	29/36 = (80.6%)
**Backes, 2014**	32/32 (100%)
**Delara, 2021**	35/37 94.6%
**Foley, 2016**	28/32 (87.5%)
**Howlett, 2024**	61/80 (76.3%)
**Jablonska, 2021**	58/58 (100%)
**Pagano, 2023**	abstract
**Sole, 2015**	21/35 (60%)
**Sprave, 2024**	Radiotherapy 8/40 (20%), Radiotherapy+Brachytherapy 15/40 (37.5%) Brachytherapy 4/40 10%
